# Knowledge, attitude and dispensing practice of the pharmacists related to complementary alternative medicines in the Riyadh region of Saudi Arabia: a cross-sectional descriptive study

**DOI:** 10.1186/s12913-022-08932-0

**Published:** 2022-12-16

**Authors:** Hanan M. Al-Yousef, Ahmad H. Alghadir, Amir Iqbal

**Affiliations:** 1 Department of Pharmacognosy, College of Pharmacy, King Saud University, Riyadh, 11433 Saudi Arabia; 2 Department of Rehabilitation Sciences, College of Applied Medical Sciences, King Saud University, P.O. Box. 10219, Riyadh, 11433 Saudi Arabia

**Keywords:** Pharmacists, Complementary alternative medicine, Knowledge, Attitude, Practice

## Abstract

**Background:**

The use of complementary alternative medicines (CAMs) has risen globally in recent times. Such medications are exclusively and readily available in the Riyadh region of Saudi Arabia through community pharmacies and other retail outlets, exposing the consumers to various risks like harmful drug interactions. These situations take pharmacists to a responsible position where they should provide evidence-based information to help consumers make safe consumption. The consumption of CAMs can be ensured safe if pharmacists have appropriate knowledge and training about their use, dosing, side effects, etc. This study aimed to investigate and evaluate the pharmacist's factual knowledge, perception and dispensing practice related to various aspects of CAMs based on gender and experience.

**Methods:**

The study followed a structured self-administered questionnaire-based cross-sectional survey design. Out of 200, with a response rate of 89.5%, 179 pharmacists (115 males; 64 females) from the Riyadh region of Saudi Arabia participated in this study. The knowledge scores of male and female pharmacists were compared using an unpaired t-test. The correlation between pharmacists’ knowledge and their work experience was determined using Pearson's correlation coefficient test, keeping the significance value at *p* < 0.05.

**Results:**

Almost all the respondents (99%) reported that they had never attended any lecture or course related to CAMs. Results show low knowledge scores, indicating poor respondents' knowledge concerning the use of CAMs. No significant difference was observed in knowledge scores based on gender, and no significant correlation between pharmacists' knowledge scores and their work experiences.

**Conclusion:**

Therefore, the study concluded that both male and female pharmacists possess equal knowledge concerning the use of CAMs, and their work experience doesn’t differentiate in their knowledge. Various factors like lack of time, etc., prevented respondents from interacting with patients. Regular organization of mandatory continuing education programs about the safe use of CAMs that can improve their knowledge is recommended. In addition, establishing a pharmacy connection network system can enhance patient monitoring and CAMs vigilance. This study lays a foundation for further work to assess pharmacists’ knowledge and practice patterns in Saudi Arabia.

**Trial registration:**

Not applicable.

## Background

With wider acceptance among consumers, use of Complementary Alternative Medicines (CAMs) has perpetuated globally in the recent times. [1–4]. According to World health organization (WHO) 70–90% of people in developing countries depend on CAMs for primary care treatment. [5, 6]. Health care in Saudi Arabia largely depends on the modern system of medicine, though traditional CAMs remain popular among patients. [7, 8]. Such medicines are exclusively and readily available in the Riyadh region of Saudi Arabia through community pharmacies, other retail outlets, and private hospitals. Though most of them are registered with the health ministry, some unregistered variants are also available in the market [9], exposing consumers to various risks, including drug interactions with modern medicines. [10]

As the sales of CAMs are on the rise, pharmacists are in responsible position to provide evidence-based information to help consumers in making safe choices about their use. [11]This can be insured only if pharmacists have appropriate knowledge and training in their use, dosing, side effects, etc. [12]It has been reported that pharmacists receive more questions from patients about the safe use of CAMs than any other medicine. [13]. Various studies around the world have reported that pharmacists have poor knowledge about these drugs [12, 14–17], and suggested that they should regularly attend continuing education programs about CAMs to update themselves [18].

To the best of our knowledge, no reported studies in the Riyadh region of Saudi Arabia assessed the difference in pharmacists' knowledge (based on gender) concerning using the CAMs, and their relation to work experience. Hence, this study aimed to determine the difference between male and female pharmacists' factual knowledge, attitude, and practice towards dispensing CAMs and the correlation between their knowledge and working experience. Results of this study would help identify perceived barriers to addressing the role of pharmacists as information providers to patients about these products.

## Methods

### Study design

The study followed a structured self-administered questionnaire-based cross-sectional descriptive design.

### Ethical considerations

The study obtained an ethical approval from the Ethics Sub-committee at King Saud University, Saudi Arabia and conducted the study in accordance with the declaration of Helsinki (2010). A signed informed consent to participate in the study was obtained from each participant prior to beginning of the study.

### Setting

The pharmacists (Male and female) working in the Riyadh region of Saudi Arabia in government/private hospitals (such as Dalla, King Salman, Al-Yamamah, Riyadh Medical, and Saudi German Hospital) and community pharmacies were screened for participation in this study. These hospitals are located distant from each other such that they receive the patients/users of CAMs from the outer and inner regions of Riyadh. The study was completed over three months, starting from April 2018.

### Study participants

**Scheme 1:**
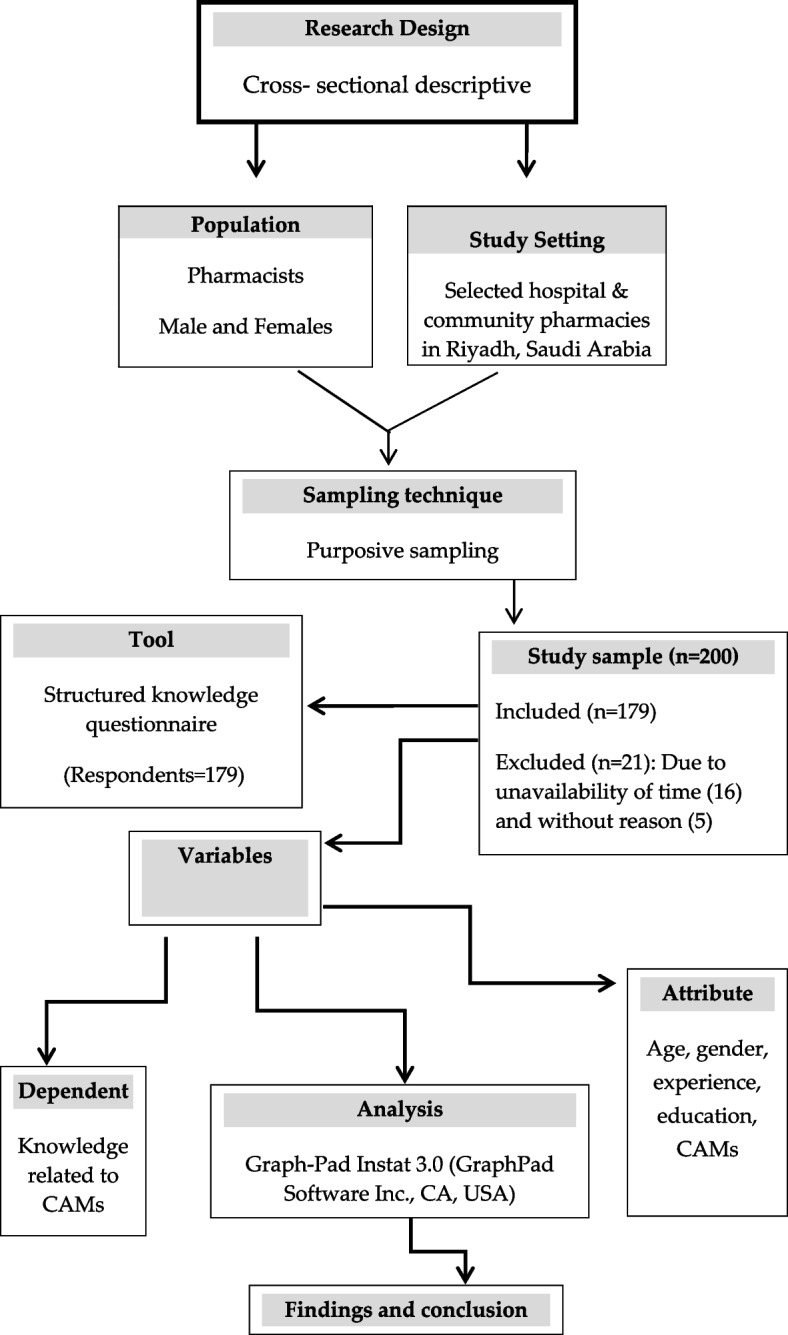
Schematic outline of research design and following procedures of the study

One-hundred seventy-nine pharmacists (male, 115; female, 64, aged between 30–35 years) were recruited from a sample of 200 based on the study’s inclusion and exclusion criteria. The participants obtained a minimum diploma certificate in pharmacy from the institutions/universities recognized by their respective countries; a license certificate authorizing dispensing the medicines from the Ministry of Health (MOH), Saudi Arabia; and a willingness to participate; were included in this study. However, the participants who did not show their diploma/degree certificates in pharmacy; pharmacists’ license certificates issued by MOH, Saudi Arabia; and showed non-cooperation in this study; were excluded from this study. A purposive sampling technique was used while listing the names of famous hospitals and community pharmacies for collecting the sample of the study. However, we randomly approached a pharmacist out of many at each place (hospital/retail pharmacy) while screening and providing the questionnaire to fill out. The licensed pharmacists working in different government and private hospitals and community pharmacies in the Riyadh region of Saudi Arabia were approached for this study. Before approaching to the study participants, an official permission was obtained from the director in respective hospitals pharmacists. They were explained about the purpose of study and their informed consent was obtained if they agreed to participate. Details of research design and following procedures of the study have been outlined scheme 1.

### Outcomes variables

The demographic characteristics of the participants were age, gender, work experience, education in pharmacy, working area (government/private hospitals, community pharmacies) and primary outcome variables were the dispensing practices related to CAMs, the attitude towards CAMs, and the knowledge about CAMs. A structured self-administered questionnaire was used for obtaining the data for the variables in this study.

### Measurements

#### Questionnaire

The structured self-administered questionnaire was: based on similar studies used for data collection. [12, 18] was used for data collection. It consisted four main categories including demographics, dispensing practices, attitudes and factual knowledge of pharmacists relevant to CAMs. With some modification, the list of commonly dispensed CAMs in Saudi Arabia (Table 5) was discussed, reviewed, and approved by a team of four experts from the department of Pharmacy. In addition, the final modified version of the questionnaire with original copy of questionnaire was also validated by another two experts (at consultant level) i.e., one from the Head of the community medicines department and another from the Head of university hospital pharmacy who also has been serving as a senior member of the Saudi Society of Clinical Pharmacy (SSCP) that represents a group of pharmacists of the type to which the questionnaire was to be administered. All questions were related to CAMs that are known to be commonly used in Saudi Arabia. The participants scored 1 for each correct answer and their knowledge score out of 10 was given on the basis of number of correct answers. Any response regarding vitamin and mineral supplement use was excluded.

Complementary alternative medicine was defined as the practices with aimed in achieving the healing effects of the products/medicines besides lacking of scientific testing or evidence from various trials, such as the use of plants (or its product) for medicinal purposes. [19]. It is traditional medicine in various parts of the world and still widely practiced today. They are sold as tablets, capsules, powders, teas, extracts, and fresh or dried plants. People use *CAMs* such as herbal supplementations trying to maintain or improve their health, revive their energy, and fitness. Many people believe that products labeled "*natural*" are always safe and good for them.

### Statistical analysis

Data was analyzed using Graph Pad Instat 3.0 (Graph Pad Software Inc., CA, USA). Mean, frequencies and percentages were used for descriptive statistics. Difference in means of knowledge scores between male and female respondents was tested by unpaired t test. Correlation between experience of pharmacists and their knowledge score was calculated by Pearson correlation coefficient. *P* value < 0.05 indicated level of significance.

## Results

### Demography

Two hundred potentially eligible pharmacists were approached, examined, and confirmed their eligibility for recruitment in this study. Out of twenty-one, sixteen participants couldn't participate due to unavailability of time, and five denied participating without reason in this study; they did not complete the questionnaire and the informed-consent form. The rest of the participants (179 with a response rate of 89.5%) signed the informed consent form and completed the questionnaire after being included in this study and their data were analyzed. Among these 64% (115) were males while 36% (64) were females. More than half of the respondents (57%) reported to be between 30 to 35 years of age and 56% respondents reported to have work experience of more than 6 years. (Table 1) Details regarding their work places have been given in Table 2.

**Table 1:** Demographic characteristics of respondents including number and percentage, (*N* = 179)

Sl. No	Variables Characteristics		Number (%) [*N* = 179]	
01	Genders	Male	115(64)	
		Female	64(36)	
02	Age Distributions (years)	< 30	38 (21)	
		30–35	102(57)	
		36–40	34(19)	
		> 40	5(3)	
03	Hospitals types	Government	63 (35)	
		Private	72 (40)	
		Comm. Pharmacy	44 (25)	
04	Experiences (years)	< 3	16 (9)	
		3–6	63 (35)	
		> 6	100 (56)	
05	Courses attended	Pharmacology	Yes 20 (11)	No 159 (89)
		Alter. Medicine		
		Phytochemistry

**Table 2:** Distribution of respondents based on their experience (years) and work places, (*N* = 179)

Experience	Community pharmacy No. (%)	Hospitals No. (%)					Total
		**Dalla**	**King** **Salman**	**Al-Yamama**	**Riyadh medical complex**	**Saudi German**	
< 3	0 (0)	4 (11)	3 (9)	5 (18)	3 (15)	2 (13)	17 (9)
3–6	20 (45)	8 (22)	13 (37)	8 (29)	9 (45)	4 (26)	62 (35)
> 6	24 (55)	25 (67)	19 (54)	15 (54)	8 (40)	9 (60)	100 (56)
Total	44 (25)	37 (21)	35 (20)	28 (16)	20 (11)	15 (8)	179 (100)

### Dispensing practices related to CAMs

40% and 35% of respondents reported that the most popular CAMs among users are green tea and tea with ginger, respectively. Other drugs that are reported to be sold are Ginkgo biloba (15%), co-enzyme Q10 (5%) and garlic (5%). In response to a question on identifying the degree of purity of CAMs, 96% reported that they could not identify it based on color, smell or ministry report. Only 13% of respondents reported keeping a record about CAMs in their departments. (Table 3).

**Table 3:** Pharmacist’s dispensing of CAMs, (*N* = 179)

Sl. No	Statement	Color	Concentrate		smell	material		Analysis in MOH		Extraction of violate oil	None
1	How can you identify the specification of the herb and the degree of purity	3 (2%)	0		0	0		3 (2%)		0	166 (96%)
2	Did you attend any CME lecture about CAMs	**No**					**Yes**				
	Number (%)	169 (99%)					2 (1%)				
3	Did you record the CAMs in your registration form in pharmacy	**No**					**Yes**				
	Number (%)	149 (86.6%)					23 (13.4%)				
4	What are the best-selling CAMs	**Green tea**	**Tea with ginger**	**Co-enzymeQ10**			***Ginkgo biloba***		**Garlic**	**Pro span**	**None**
	Percentage	40	35	5			15		5	1	0

### Attitude towards CAMs

Around 33% of the respondents reported that they gave customers the required information and advice as they inquired while dispensing them. Only 4% of respondents reported giving their opinion about the impediments discussed with the patient. (Table 4).

**Table 4:** Pharmacist attitude toward complementary alternative medicines (CAMs), (*N* = 179)

Sl. No. Statements		No	Yes				
1	Do you counsel your customers about using of CAMs?	116 (67%)	58 (33%)				
			No. (%)	SE	How to use	How long	Others
				2 (1%)	3 (2%)	0 (0)	144 (97%)
2	What are the impediments to discuss CAMs with the patient from your point view?	Time constrains	Lack of Focus	Lack of interest of the patient	Lack of leaflet contains information	Lack of adequate studies that include CAMs	None
		1 (1%)	1 (1%)	3 (2%)	1 (1%)	1 (1%)	172 (96%)

### Knowledge about CAMs

Low knowledge scores indicate the poor knowledge of respondents concerning the use of CAMs. (Table 5) Almost all the respondents (99%) reported that they had never attended any CME lecture or course related to CAMs after getting registered as pharmacists. (Table 3) However, 54% of respondents reported that scientific books were their most favored source of information about CAMs besides the study course lecture, internet, scientific journals, drug leaflets, pamphlets, brochures, and others. (Table 6).

**Table 5:** Pharmacist’s knowledge about commonly dispensed CAMs in Saudi Arabia pharmacies

Sl. No	The text of the multiple-choice questions served to all participants	Pharmacists' Response Number (%)	
		**Incorrect**	**Correct**
1	Herbal product that may lower blood cholesterol level	160 (93%)	12 (7%)
2	Herbal product that increases bleeding effect when used with anticoagulants	69 (40%)	103 (59%)
3	Herbal product that may cause insomnia and anxiety	38 (21%)	136 (78%)
4	*Black Cohosh* is used for	45 (25%)	129 (72%)
5	*Echinacea* is used for	99 (57%)	75 (43%)
6	*Valerian* root should not be used with drugs that treat	105 (60%)	69 (40%)
7	Which the following is appropriate information for the patient who is considering the use of *Ginkgo biloba*	49 (28%)	124 (72%)
8	Which the following side effects have been linked to Ephedra/Caffeine	89 (51%)	84 (49%)
9	From the following drug, the drug that treated hypertension	56 (32%)	118 (68%)
10	In patients who have a prior history of heart disease, preparation that protect from heart attack	84 (49%)	89 (51%)
11	St John Wort is used to treat	70 (40%)	104 (60%)
12	Enlarged Prostate is common male disease, the herb used to treat	98 (57%)	75 (43%)
13	*Chrysanthemum* used to treat Migraine Headache, the common name of this herb	25 (14%)	148 (85%)
14	*Eucalyptus* is used for	46 (27%)	127 (73%)
15	*Marjoram* has many uses, the common name of this herb	80 (46%)	93 (54%)
16	Alfalfa active ingredient	25 (14%)	149 (86%)

**Table 6:** Pharmacists' source of information about the herbal drugs derived from plant origin (*N* = 179)

Number (%)	Sources of information	Sl. No
1	Study Course Lectures	20 (11)
2	Scientific Books	96 (54)
3	Internet	35 (19)
4	Scientific journals	5 (3)
5	Delegate advertising	0 (0.0)
6	Drug leaflet	7 (4)
7	Others	16 (9)

### Correlation between pharmacist’s knowledge and their work experience

**Fig. 1:**
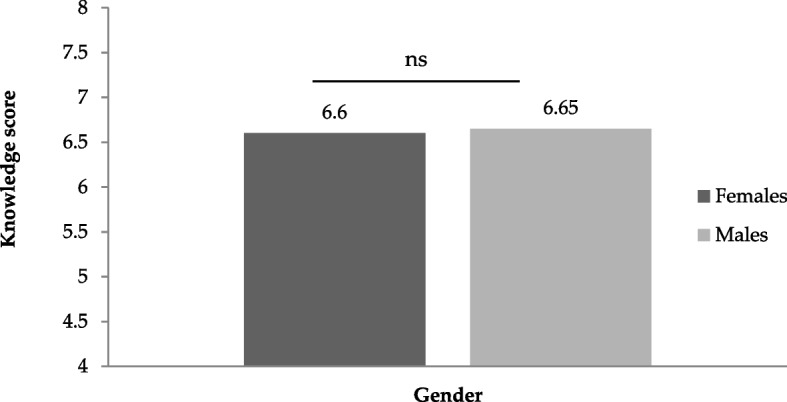
Comparison of respondent’s knowledge scores based on their gender. Note the insignificant (ns) difference between male and female respondents (*P* = 0.912)

**Fig. 2:**
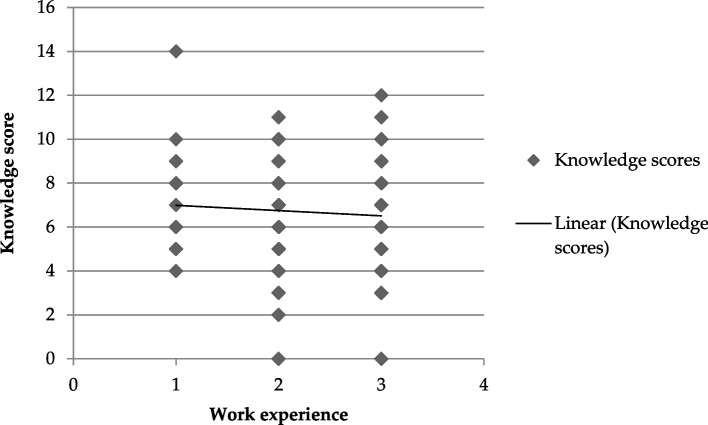
Relation between respondent’s years of work experience and their knowledge towards herbal products. Note the insignificant correlation (*P* = 0.377) experience and knowledge score (KS)

There was no significant difference in pharmacist’s knowledge scores based on gender (*P* = 0.912) and no correlation of their knowledge with their work experience (*P* = 0.377) (Tables 7, 8; Figs. 1, 2).

**Table 7:** The relationship between gender and mean knowledge score (± SD) of respondents

Gender	Number (%)	Average knowledge score (Mean ± SD)
Female	64 (36)	6.60 ± 2.61
Male	115 (64)	6.65 ± 2.28
**Total**	**179 (100)**

**Table 8:** Correlation between work experience (years) and mean knowledge score (± SD) among respondents (*N* = 179)

Experience	Number (%)	Average knowledge score (Mean ± SD)
< 3	17 (9)	7.41 (2.39)
3–6	62 (35)	6.51 (2.51)
> 6	100 (56)	6.58 (2.32)
**Total**	**179 (100)**	**6.83 (2.40)**

## Discussion

With a response rate of 89%, the sample size of this study gives a fair representation of pharmacists working in various hospitals (government and private) and community pharmacies across the Riyadh region in Saudi Arabia. This study aimed to determine the knowledge, attitude and dispensing practice of both male and female pharmacists towards CAMs in the Riyadh region of Saudi Arabia and to establish the correlation between their knowledge and their years of working experience as pharmacists. Results show that although CAMs are readily available in the country, both male and female pharmacists have poor knowledge about their use. Also, no correlation existed between the knowledge about using CAMs and the work experiences as pharmacists.

Studies have shown increasing popularity of CAMs in Saudi Arabia, with at least 24% of patients attending a healthcare center reporting to use it sometime in their lifetime. [20]. Although users use CAMs without informing their physicians, diabetic patients have been found to be among highest users of them. [21, 22]. The belief that CAMs are more natural than modern medicines which are chemicals, [23] make it more popular among patients. In addition, the patient believes that licensed pharmacists are easily accessible to patients and does provide more accurate and authentic information about various aspects of CAMs.

Harmful drug interaction has been reported in patients using multiple medications, especially when CAMs are consumed along with modern medicines without proper consultation. [23, 24]. Pharmacists should be aware of such interactions so that they can alert patients if they are taking multiple medications including these. Our results show that only 33% of the respondents reported to counsel patients while dispensing drugs. A previous similar study conducted on male pharmacists in the Asir region of Saudi Arabia, declared that 36% respondents counsel the patients while dispensing the drugs. [25]. Pharmacists should inquire patients about their medications and should be informed about possible side effects and adverse medication interactions with the additional use of CAMs. Continuing medical education (CME) lectures that provide information on safety, purity and possible side effects about various CAMs should be organized for pharmacists regularly [18]. Besides this pharmacies can be provided with printed materials about harmful effects of such drug and its possible interaction with other drugs that can be given to patients while dispensing them.

Although more than half of the respondents verbally reported that they have good knowledge about CAMs, however, failed in knowledge testing related to CAMs. Thus, the contention between self-rating and the actual test results of their knowledge can affect security of patients. This is concurrent with results of other similar studies. [26]. In contrast, a previous study revealed the knowledge of pharmacists was good to excellent (84%) towards various aspects of herbal products in the Asir region of Saudi Arabia [25]. Our results also show that there is statistically insignificant difference between knowledge score based on gender and work experience. Similar results have been shown in neighboring regions of Oman and Palestine. Although not subjectable to statistical testing, crude comparison of the actual knowledge of respondents regarding CAMs show higher scores in Oman while lower scores in Palestine in all domains as compared to results of this study [27, 28].

The percentage of pharmacists reporting either discussing with patients about CAMs while dispensing or documenting their information in the record is low. This could be due to pharmacists’ low self-confidence due to poor knowledge or hesitation in explaining to inquiry about it or lack of time. There is need for improvement in pharmacist’s documentation by implementing clinical pharmacy and pharmaceutical care in pharmacy practice, which allows them to record all information about patient’s medications. [9, 25, 29]

This study reveals a set of impediments that limit pharmacists from discussing CAMs with patients while dispensing. Lack of time, authoritative resources, scientific evidence, knowledge, and interest are few of them. These have been previously being identified in similar studies. [29–31]. Internet has been shown to be popular source of information among respondents in comparison to lectures, pamphlets and brochures. Hence, it is worthy to assess the electronic resources before utilizing them for reliability, precision, reputability and profundity of data. Various reference books about evaluation of CAMs are available that can serve as a good guide for health care professionals. [32–34]

Implications for practice.

Revealing the pharmacists’ knowledge of various aspects of the CAMs as low will catch the attention of the health ministry, hospitals, and community pharmacies’ management person/owners for working towards improving pharmacists' knowledge on CAMs through various educational programs/CME/activities. The insignificant difference between male and female pharmacists’ knowledge of CAMs will provide an equal opportunity and confidence for the female pharmacists who were qualified and recently started working in pharmacy parallel to male pharmacists in Saudi Arabia. The result will also provide an equal opportunity for the newer pharmacists working parallel to the senior pharmacists in the pharmacy outlets.

## Limitations of study

The sample obtained for the current study was limited to the Riyadh region of Saudi Arabia, though the study was based on a cross-sectional survey design. This study could be repeated on a larger sample in various parts of the country with equal representations from both genders and analyzing more demographic characteristics of the participants for better representation of results. The study did not follow the randomization method while obtaining the study's sample. Future research should follow the randomization method while obtaining the desired sample to achieve the results approximate to the entire population. The study was also limited in using a Chi-square test for categorical analysis of the demographic variables except for gender which might reveal the dependency or extent of relation over each other. A future study should take on these points for getting better analysis and output for the readers.

## Conclusions

Therefore, the study concluded that both male and female pharmacists possess equal knowledge concerning the use of CAMs, and their work experience doesn’t differentiate in their knowledge. Various factors like poor knowledge, lack of time, hesitation to discuss, interest, etc., prevented respondents from interacting with patients. Regular organization of mandatory continuing education programs about the safe use of CAMs that can improve their knowledge is recommended. In addition, establishing a pharmacy connection network system can enhance patient monitoring and CAMs vigilance. This study lays a foundation for further work to assess pharmacists’ knowledge and practice patterns in Saudi Arabia.

## Data Availability

The datasets used and/or analyzed during the current study are available from the corresponding author on reasonable request.

## Abbreviations

CAMs: Complementary Alternative Medicines
KAP: Knowledge, Attitude, and Practice of dispensing CAMs
